# Takotsubo Syndrome and Arrhythmias: The Chicken and Egg Dilemma

**DOI:** 10.7759/cureus.99423

**Published:** 2025-12-16

**Authors:** Ghita Bennis, Samy Lebbar, Sara Hafid, Fatimazahra Merzouk

**Affiliations:** 1 Cardiology, Cheikh Khalifa International University Hospital, Mohammed VI University of Health Sciences (UM6SS), Casablanca, MAR

**Keywords:** cardiac mri, complete atrioventricular block, implantable cardioverter-defibrillator, takotsubo syndrome, torsades de pointes (tdp)

## Abstract

Takotsubo syndrome (TTS), also known as broken heart syndrome, is a transient and reversible cardiomyopathy characterized by left ventricular dysfunction in the absence of obstructive coronary artery disease. Although often considered benign, TTS can lead to serious arrhythmic complications such as atrioventricular block (AVB) and life-threatening ventricular arrhythmias.

We present the case of a 74-year-old woman with cardiovascular risk factors who developed complete AVB and torsades de pointes during the course of a TTS episode. The diagnosis was based on clinical presentation and imaging findings, including recovery of left ventricular ejection fraction. Cardiac magnetic resonance imaging was limited by technical constraints but supported the diagnosis.

This case highlights the complex arrhythmic manifestations that may accompany TTS and underscores the importance of careful rhythm monitoring and device selection. Implantable cardioverter-defibrillator therapy was ultimately chosen due to the occurrence of sustained ventricular arrhythmias.

Clinicians should be aware that TTS, though reversible, may present with conduction disturbances and ventricular arrhythmias requiring aggressive management and appropriate device implantation strategies.

## Introduction

Takotsubo syndrome (TTS) is a transient stress-induced cardiomyopathy characterized by acute, reversible left ventricular (LV) dysfunction, typically a transient apical ballooning of the LV, in the absence of obstructive coronary artery disease [1-3].

Although LV dysfunction is usually reversible, some rhythm disturbances occurring during the acute phase can be life-threatening and pose significant therapeutic challenges. These include ventricular tachycardia and fibrillation, torsades de pointes, atrial fibrillation, sinus node dysfunction, and complete atrioventricular block (AVB) [2,4,5].

An additional challenge lies in the fact that arrhythmias in TTS may either precede and trigger the syndrome or arise as a consequence of it, a bidirectional relationship that remains an area of clinical uncertainty.

We report the case of a patient who presented with TTS complicated by several of these arrhythmias.

## Case presentation

The patient was a 74-year-old woman with several cardiovascular risk factors, including type 2 diabetes, hypercholesterolemia, obesity, and advanced age.

Her symptoms began 15 days prior to hospital admission with the onset of dyspnea classified as New York Heart Association (NYHA) functional class III, accompanied by asthenia and dizziness following an emotional stressor (a family argument). Thirteen days later, the development of multiple syncopal episodes prompted medical consultation. Serum troponin levels were elevated.

**Figure 1:**
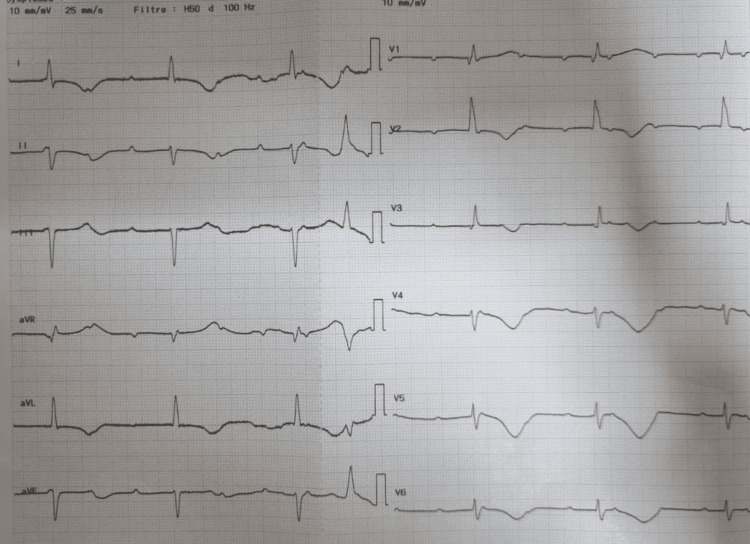
Admission electrocardiogram on admission showing complete atrioventricular block with a ventricular escape rhythm at 35 bpm and marked QTc prolongation

Upon admission, physical examination revealed a bradycardia of 35 beats per minute (bpm), a blood pressure of 150/70 mmHg, and an oxygen saturation of 96% on room air. The electrocardiogram (ECG) showed a complete AVB with a Bazett-corrected QT interval (QTc) of 562 ms (Figure 1). The patient underwent temporary transvenous pacing, and coronary angiography revealed only non-significant coronary atherosclerosis.

A transthoracic echocardiogram (TTE) performed after stabilization showed hypokinetic cardiomyopathy with severe LV systolic dysfunction. The left ventricular ejection fraction (LVEF) was estimated at 35%, with akinesia of the apical and adjacent mid-ventricular segments, contrasting with compensatory basal hyperkinesia, raising suspicion for TTS. The calculated InterTAK Diagnostic Score was 79.8%. Due to the patient's renal impairment, ventriculography was not performed.

A first cardiac magnetic resonance imaging (MRI) examination was attempted shortly after admission, including cine steady-state free-precession (SSFP) sequences, T2-weighted imaging, and early and late gadolinium enhancement (LGE) sequences. However, image quality was markedly degraded by susceptibility artifacts from the temporary pacing lead, and severe bradycardia after lead removal caused repeated mis-gating. As a result, the LGE assessment was incomplete, and no reliable tissue characterization could be obtained.

**Figure 2:**
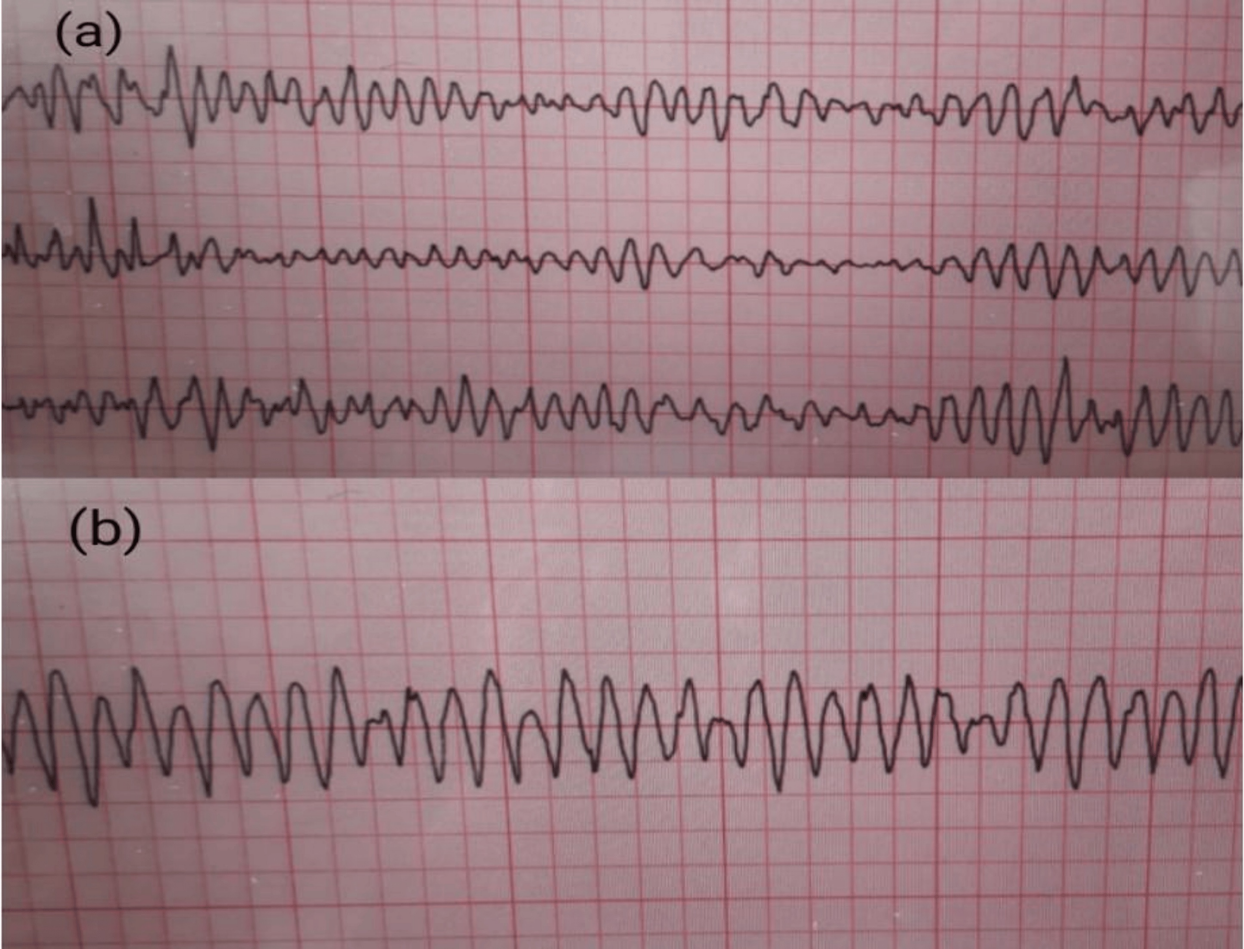
Rhythm tracing during follow-up MRI demonstrating torsades de pointes degenerating into ventricular fibrillation during the removal of the temporary pacing lead (a) Torsades de pointes. (b) Ventricular fibrillation. MRI: magnetic resonance imaging

Approximately a week later, a follow-up MRI was again attempted, requiring the removal of the pacing system; however, the resulting profound bradycardia precipitated a convulsive syncope, which rapidly evolved into torsades de pointes and subsequently degenerated into ventricular fibrillation (Figure 2). She was successfully resuscitated. Notably, her electrolyte levels were within normal limits.

**Figure 3:**
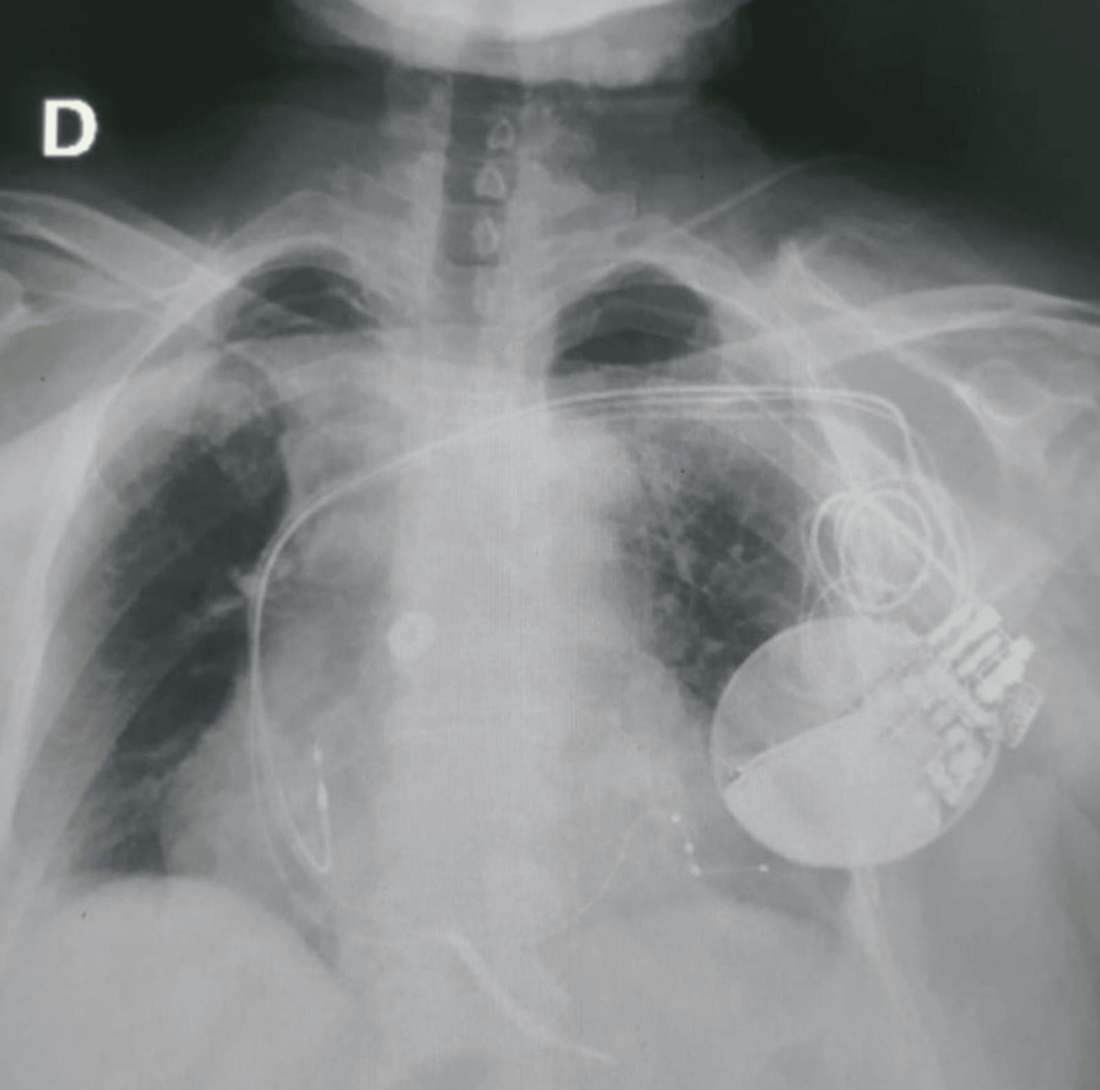
Chest radiograph showing the appropriate positioning of the cardiac resynchronization therapy defibrillator leads (RA, RV, and LV via the coronary sinus) 24 hours after implantation RA: right atrium; RV: right ventricle; LV: left ventricle

A cardiac resynchronization therapy defibrillator device (CRT-D device) was subsequently implanted (Figure 3). Heart failure therapy was maintained, and beta-blockers were initiated and progressively optimized.

The course of the cardiomyopathy was characterized by partial recovery, with improvement of LVEF to 40% by day 15.

## Discussion

TTS is generally considered a benign cardiomyopathy. However, some of its complications can be life-threatening, including cardiogenic shock, severe arrhythmias, ventricular thrombus formation, ventricular septal defect, or LV free wall rupture [6].

The association between TTS and arrhythmias is relatively uncommon. Reported prevalence rates include 3.4% for ventricular arrhythmias, 4.7% for atrial fibrillation, 4.3% for conduction abnormalities, and 2.9% for AVB [5].

The precise pathophysiological mechanism of TTS remains uncertain. However, stress-induced catecholamine excess is widely accepted as a potential trigger. Sympathetic nervous system activation leads to the adrenergic stimulation of the myocardium, resulting in transient changes in electrophysiological properties and contractility [7]. The acute phase is therefore characterized by cardiac adrenergic dysfunction, which typically resolves over weeks to months.

In our patient, a first consideration concerns the diagnostic value of cardiac MRI in TTS. Although some studies suggest that MRI is a key tool for the noninvasive evaluation of TTS, offering detailed anatomical and tissue characterization with high reproducibility [8], it was of limited use in this case due to image artifacts caused by the temporary pacing lead and severe bradycardia following its removal. The diagnosis was therefore made based on clinical presentation and high InterTAK score, in addition to the observed improvement in LVEF.

A second major question concerns the temporal and causal relationship between TTS and AVB. It can be interpreted through three main mechanisms. First, some authors suggest that complete AVB may itself act as a physiological stressor, triggering a catecholamine surge capable of precipitating TTS [9]. In our patient, the initial presentation was dominated by recurrent syncope and profound bradycardia occurring several days before the diagnosis of TTS, with no prior history of conduction disease. This sequence, combined with the high InterTAK score and the absence of basal wall-motion abnormalities near the atrioventricular node, makes this mechanism particularly plausible. On the other hand, it has also been proposed that TTS may itself induce AVB, although this usually involves basal ventricular edema or dysfunction affecting the conduction system [10], features that were not observed here. Finally, while a purely coincidental association between the two entities cannot be completely excluded, it appears less likely given the tight temporal link between the conduction disturbance and the subsequent development of apical ballooning. Taken together, the clinical timeline and imaging findings strongly suggest that complete AVB preceded and triggered TTS in this case.

This distinction is clinically important, as it influences management decisions. Not all patients with TTS and AVB require permanent pacemaker (PM) implantation. According to current evidence, PM implantation is indicated when AVB is clearly the trigger for TTS, while other cases should be evaluated individually [11]. Moreover, when AVB is believed to result from TTS, the optimal timing for PM implantation remains controversial, especially given the infection risk associated with prolonged temporary pacing [12].

Persistent AVB beyond the acute or subacute phase has been reported in several cases during follow-up and device interrogation [13,14]. These observations support PM implantation in patients presenting with AV conduction disturbances at initial presentation.

In contrast, for ventricular arrhythmias, some authors recommend temporary management using wearable cardioverter-defibrillators (WCDs) until LV function and repolarization normalize [12,13,15]. For instance, in a similar case, both a PM and a WCD were initially used, with an implantable cardioverter-defibrillator (ICD) implanted later based on early device monitoring [16]. A final consideration concerns the choice of ICD type, particularly in pacing-dependent patients, in whom right ventricular (RV) asynchrony may further impair LV function. Indeed, chronic cardiomyopathy induced by long-term RV pacing has been well documented [16].

The patient demonstrated near-complete pacing dependence after admission, indicating an anticipated >90% RV pacing burden. Given the reduced LVEF (35%) and the likelihood of chronic RV pacing worsening LV function, device choice followed the 2021 European Society of Cardiology (ESC) pacing guidelines, which recommend biventricular pacing in patients with high-grade AVB and LVEF <40% (Class I, Level A) [17]. CRT-D was therefore selected to prevent pacing-induced cardiomyopathy and to provide defibrillation capability in a patient who had already experienced malignant ventricular arrhythmias, especially since WCDs were not available in our setting.

## Conclusions

TTS may be associated with a wide spectrum of arrhythmias, ranging from tachyarrhythmias to conduction disturbances, thereby presenting multiple diagnostic and therapeutic challenges.

In the present case, complete AVB manifested with syncope and was followed several days later by torsades de pointes and ventricular fibrillation, from which the patient was successfully resuscitated.

Early implantation of a permanent PM may be necessary in cases of syncopal AVB, particularly when spontaneous recovery of conduction is unlikely. In contrast, ventricular arrhythmias may be transient and reversible, raising the clinical dilemma between implanting a defibrillator versus opting for temporary management or close follow-up.
